# Sociodemographic and lifestyle factors and the risk of metabolic syndrome in taxi drivers: A focus on street food

**DOI:** 10.3389/fnut.2023.1112975

**Published:** 2023-02-23

**Authors:** Machoene Derrick Sekgala, Maretha Opperman, Buhle Mpahleni, Zandile June-Rose Mchiza

**Affiliations:** ^1^School of Public Health, University of the Western Cape, Bellville, South Africa; ^2^Human and Social Capabilities, Human Sciences Research Council, Cape Town, South Africa; ^3^Functional Food Research Unit, Department of Biotechnology and Consumer Science, Cape Peninsula University of Technology, Cape Town, South Africa; ^4^Non-Communicable Diseases Research Unit, South African Medical Research Council, Cape Town, South Africa

**Keywords:** street food, metabolic syndrome, male taxi drivers, physical activity, socio-economic status, South Africa, waist circumference

## Abstract

**Background:**

In South Africa, similar to other populous countries, the taxi industry is an important form of transportation that contributes to the country's development. As a result, minibus taxi driving is an occupation characterized by strenuous activities such as long hours of driving, limited rest, and challenges related to securing passengers, among several others. Consequently, to combat stress, some commercial drivers resort to smoking, overeating unhealthy food sold at transportation interchange areas (i.e., taxi ranks), and participating in sedentary behaviors. Most of these activities are risk factors for metabolic syndrome (MetS).

**Aim:**

Therefore, this study aimed to investigate the sociodemographic and lifestyle factors that predispose South African taxi drivers who work in the Cape Town Metropole area to the risk of developing MetS.

**Methods:**

This cross-sectional study used a convenient sampling method that included 185 male minibus taxi drivers aged 20 years or above. The participants were interviewed using a validated questionnaire to gather information regarding their sociodemographic characteristics and lifestyle practices. They also underwent physical and metabolic assessments, and the International Diabetes Federation (IDF) criteria were used to diagnose people with MetS.

**Results:**

Overall, the mean age and driving experience of the taxi drivers were 40.0 years (SD: 10.7) and 9.1 years (SD: 7.4), respectively, with those with MetS being significantly older and having more driving experience than those without. Older participants were 3 and 2.9 times more likely to be diagnosed with MetS than the younger participants. Most taxi drivers (70%) met the IDF diagnostic criteria for MetS. Smokers, those who spent more than 100 ZAR (USD 5.9) and those who spent less than 1.4 MET-minutes per week on physical activity were 1.96, 2.0, and 13.6 times more likely to suffer from MetS that those who were nonsmokers, those who spent less than 100 ZAR and those who spent <1.4 MET-minutes per week on physical activity. Consumption of alcohol and sugar-sweetened beverages (SSBs), as well as takeaway and fried foods, snacks, and sold by the SF vendors, increased the likelihood of developing MetS, abnormal HDL-C, TG, and hypertension, while avoiding takeaway and fried foods decreased this likelihood. Taxi drivers who also avoided consuming fresh fruits had abnormal HDL-C.

**Conclusion:**

These findings have significant public health implications, highlighting the need for South African policymakers to adopt a system-level approach to promote lifestyle changes among taxi drivers within the taxi industry. This can help reduce the health risks faced by these drivers and improve their overall health profile.

## Introduction

Several international epidemiological studies have found the prevalence of metabolic diseases to be high among occupational drivers compared to other professionals, such as industrial and office workers (1–3). For example, the majority of professional drivers are at an increased risk of hypertension, myocardial infarction, and hemorrhagic stroke (4). Furthermore, most drivers are in the habit of eating large main meals and consuming snacks (often oily and fried) and fast-food items from street vendors between trips. In addition, many of them resort to alcohol and smoking to overcome stress. It follows logically that they may have an additional risk of developing metabolic diseases. According to Kurosaka et al. (5), taxi driving is characterized by poor eating habits, ongoing stress from driving, and exposure to various health hazards such as air pollution and a lack of physical activity.

In South Africa, taxi drivers and commuters are major consumers of street food (SF) since it is relatively cheap and easily accessible at taxi ranks and bus stations (6, 7). According to Mchiza et al. (8) and Hill et al. (9), the food sold in the streets of Cape Town and surrounding areas seems to be a public health risk since it is energy-dense and high in saturated fat, trans fats, salt, and sugar. Taxi drivers working in these areas may be at risk of developing metabolic syndrome (MetS) as they have been identified to be among the 38% of individuals who consume SF almost daily (6).

Good health is a basic constitutional right for all South African citizens (10). The Occupational Health and Safety Act (OHSA) Section 12(C) (11) requires medical surveillance for all individuals who have high-risk occupations, such as taxi drivers. Similar to other countries, the taxi industry is an important form of transportation in South Africa, contributing to the country's development (12). However, less focus has been given to this industry to ensure that its workers are in good health. To the best of our knowledge, there has never been any health intervention directed at improving the health condition of taxi drivers in South Africa. Substantiated evidence (13–18) suggests that a healthy lifestyle, including healthy eating and regular physical activity, can help to reduce weight, reduce blood pressure, and improve lipid disorders, including raising high-density lipoprotein cholesterol (HDL-C) and lowering triglycerides (TGs). Moreover, unhealthy eating habits and a sedentary lifestyle are known as modifiable risk factors for MetS among taxi drivers (19).

To our knowledge, to date, there are no data on lifestyle and SF consumption in relation to metabolic syndrome (MetS) among minibus taxi drivers in the Western Cape, South Africa. The current study is the first of its kind in South Africa since it investigated the understudied population of minibus taxi drivers, examining their biochemical parameters, sociodemographic characteristics, and lifestyle practices, with a particular focus on SF consumption and the association of these factors with MetS and its components. The results of this study provide valuable insights for further public health research in this neglected field. Moreover, it will contribute to developing targeted interventions to curb the escalation of MetS in adult male South Africans, especially those working in long-duration driving business.

## Materials and methods

### Study participants and sampling size

This cross-sectional study was conducted among 185 professional taxi drivers, who were recruited from taxi ranks in Bellville and Cape Town. They were at least 20 years old. This study used a convenient sampling method, and its aim was not to make generalizations about the entire population but rather to focus on taxi drivers who consume SF. These taxi ranks were chosen because they are the two major transport interchange hubs in the Cape Metropolitan Area in South Africa's Western Cape Province. Some of the data used in this study were used in a previous paper (20). The detailed sample size selection, including the power sampling calculation for the current study, is presented elsewhere (20). The participants of this study were full-time minibus taxi drivers, who had been working in this field for at least 1 year and consumed SF at least three times per week. They donated blood samples that were analyzed in a laboratory to diagnose the presence of MetS. We excluded taxi drivers who had a history of non-communicable diseases (NCDs) such as hypertension, kidney failure, hypo- or hyper- thyroidism, liver diseases, known cardiovascular diseases (CVDs), or diabetes mellitus since their eating habits might have been changed based on the advice given by their health practitioners.

### Data collection

#### Data on sociodemographic and lifestyle practices

A previously validated and structured questionnaire developed and validated for use in South Africans aged 15 years and older, which was successfully used in the first South African National Health and Nutrition Examination Survey (SANHANES-1) (21), was administered by a trained researcher to collect data on sociodemographic characteristics (i.e., age, marital status, race, and education level) and lifestyle practices (i.e., physical activity levels, alcohol consumption, and cigarette smoking) from the taxi drivers *via* face-to-face interviews. Moreover, the duration of sleep, driving experience, and money spent on purchasing SF were assessed using a validated questionnaire used in the study by Hill et al. (6).

The International Physical Activity Questionnaire (IPAQ) (22) was also used to measure the level of physical activity (PA). The results were then based on the calculated physical activity levels (PAL) using the MET-minutes per week criteria. In this case, a sedentary lifestyle was regarded as PAL < 1.4 MET-minutes per week, with low being PAL between 1.4 and 1.69 MET-minutes per week, moderate being PAL between 1.7 and 1.9, and vigorous being PAL ≥ 2 (23).

#### Frequency of consuming street food

The SANHANES-1 questionnaire (21) was also used to collect information regarding the frequency of consuming street food (FF). The FF list comprised processed meat (i.e., sausages, polony, and cold cuts, such as Viennas, Frankfurters, Russians, and salami); fast food/takeaway foods, including pizzas, fried chicken, fried fish, and burgers, that were packaged to take home; fried meat and fish dishes (i.e., chips, fried chicken, and fried fish) that were consumed on site; deep-fried snacks (i.e., fries/chips, vetkoek, samoosas, and doughnuts), fresh fruits (i.e., all kinds of fruits, excluding fruit juices and dried fruits), sugar-sweetened beverages (SSBs) (i.e., gas/fizzy and reconstituted cold drinks). Consumption frequency for each food item was measured as “none”, “every day”, “1–3 times per week”, and “4–6 times per week”.

#### Anthropometric measurements

A nonelastic tape was used to measure the waist circumference (WC) at the narrowest point between the lower rib and the upper iliac crest. A cut-off point of ≥94 cm was used to determine abnormal WC levels in men (24).

#### Blood pressure

After the participant had been seated for 5 min or longer, three blood pressure (BP) readings were taken from the right arm in a sitting position using an electronic Micronta monitoring kit (25). Normal systolic BP (SBP) was regarded as a BP that was ≤ 130 mmHg or a diastolic BP (DBP) that was ≤ 85 mmHg (24).

#### Biochemical parameters

The fasting blood glucose (FBG) was estimated using the capillary method with a glucometer (OneTouch^®^). To measure biochemical parameters, a venous fasting blood sample was obtained. The plasma lipid profile was used for MetS analysis. The concentration of triglycerides was assessed using the phosphoglycerides oxidase peroxidase method, while the HDL-C was analyzed using the colorimetric non-precipitation method. The IDF criterion was used to diagnose MetS (26). According to the IDF definition, abdominal obesity (i.e., an abnormal WC reading) and two or more of the other four metabolic risk factors are required to diagnose MetS. The cutoff points for the five MetS risk factors are as follows: WC ≥94 cm for men; TG ≥ 1.7 mmol/l; SBP ≥ 130mmHg or DBP ≥ 85 mmHg; FBG ≥ 5.6 mmol/l; and HDL-C < 1.0 mmol/l.

### Statistical analysis

Descriptive statistics were used to describe the basic features such as the categories, distribution, and spread of metabolic status, dietary intake, and lifestyle practices using sociodemographic characteristics. In this case, data were analyzed using the analysis of variance (ANOVA) and the Kruskal–Wallis tests and presented as frequencies, means, medians, and standard deviations, depending on whether they were categorical or continuous. The associations between different variables were analyzed using the Chi-square test. A binary logistic regression analysis was conducted to examine the odds ratios (OR). Multivariate analyses using multiple logistic regression models, which incorporated all risk factors for MetS while adjusting for the effect of possible confounders such as age, employment status, marital status, ethnicity, physical activity, and monthly income, were also applied (AOR). 95% confidence intervals (CIs) that did not overlap and *p*-values that were less than 0.05 indicated significant differences and associations between variable results. All data were analyzed using the statistical package for social sciences (SPSS version 28.0 for Windows; SPSS Inc., Chicago, IL, USA).

## Results

Table 1 presents the sociodemographic characteristics of the study participants based on their MetS status. Overall, the mean age of the participants was 40.0 years (SD: 10.7), with those suffering from MetS being significantly older than those without. There were 10.2% more participants within the age group of 20–39 years. There was a significantly higher prevalence of participants with MetS in the older age group than in the younger age group (61 vs. 39%).

**Table 1:** The distribution of sociodemographic characteristics of South African minibus taxi drivers by metabolic syndrome status.

	***N* = 185**	**IDF MetS**		***P* value**
		**No (*****n*** = **108)**	**Yes (*****n*** = **77)**	
**Age_(years)**				
M ± SD	40.0 ± 10.7	37.3 ± 10.2	43.7 ± 10.3	< 0.001
**N(%)**				
20-39	102 (55.1)	72 (66.7)	30 (39.0)	
≥40	83 (44.9)	36 (33.3)	47 (61.0)	< 0.001
**Ethnicity**				
**N(%)**				
Black	146 (78.9)	85 (78.7)	61 (79.2)	
Non-Black	39 (21.1)	23 (21.3)	16 (20.8)	0.932
**Level of Education** ***N*** **(%)**				
No schooling/primary education	58 (31.4)	35 (32.4)	23 (29.9)	
Some high school/high education	127 (68.6)	73 (67.6)	54 (70.1)	0.714
**Marital status**				
***N*** **(%)**				
Single/separated/divorced	97 (52.4)	60 (55.6)	37 (48.1)	
Married/living as married	88 (47.6)	48 (44.4)	40 (51.9)	0.314
**Driving experience (years)**				
M ± SD	9.1 ± 7.4	7.2 ± 6.1	11.7 ± 8.4	< 0.001
**N(%)**				
1–7	103 (55.1)	72 (66.7)	31 (40.3)	
≥8	82 (44.3)	36 (33.3)	45 (59.7)	< 0.001

IDF, International Diabetes Federation; MetS, metabolic syndrome.

While there were no other significant differences in sociodemographic characteristics in relation to MetS in this cohort, the mean driving experience of the participants was 9.1 years (SD: 7.4). In this case, the participants who presented with MetS had significantly higher driving experience compared to those without. There was also a significantly higher prevalence of participants with MetS who had a driving experience of 8 years or more compared to those with a driving experience of one to seven years (59.7 vs. 40.3%).

Table 2 shows that older participants were 3 and 2.9 times more likely to have MetS than younger participants. While the significant association of MetS with age was unavailable when the data were adjusted for lifestyle practices (i.e., cigarette smoking, alcohol consumption, sleeping duration, physical activity level, and money spent on SF each day), it was available for age after we removed the confounding effects of the other sociodemographic variables explored in the current analysis.

**Table 2:** A binary logistic regression analysis to show the association between the sociodemographic characteristics and the MetS status of South African minibus taxi drivers.

	**IDF Metabolic syndrome**											
				**Model 1**			**Model 2**			**Model 3**		
	**Crude**	**95% CI**	***P*** **value**	**AOR**	**95% CI**	***P*** **value**	**AOR**	**95% CI**	***P*** **value**	**AOR**	**95% CI**	***P*** **value**
**Age (years)**												
20–39	1			1			1			1		
≥40	3.133	1.706–5.756	< 0.001	0.541	0.217–1.351	0.188	0.589	0.225–1.539	0.280	2.955	1.2955–6.969	0.013
**Ethnicity**												
Black	1			1			1			1		
Non-Black	0.969	0.473–1.987	0.932	0.908	0.356–2.312	0.839	0.991	0.378–2.597	0.986	0.560	0.245–1.280	0.169
**Level of Education**												
No schooling/primary education	1			1			1			1		
Some high school/high education	1.126	0.598–2.120	0.714	2.880	1.212–6.847	0.017	2.676	1.103–6.506	0.030	2.004	0.940–4.273	0.072
**Marital status**												
Single/separated/divorced	1			1			1			1		
Married/living as married	1.351	0.752–2.429	0.314	0.557	0.254–1.219	0.143	0.639	0.282–1.450	0.284	0.893	0.449–1.778	0.748

OR, odds ratio; AOR, adjusted odds ratio. Model 1: adjusted for Cigarette smoking, Physical Activity Level and Money spent on Street Food each day; Model 2: adjusted for Cigarettes smoking, Alcohol drinking, sleeping duration, Physical Activity Level and Money spent on Street Food each day; Model 3: adjusted for age ethnicity, level of education, marital status and driving experience.

When examining the components of MetS (Table 3), the overall mean values for WC, TG, HDL-C, SBP, DBP, and FBG were 99.1 (SD: 18.3), 1.3 (SD: 1.1), 1.1 (SD: 0.3), 133.4 (SD: 17.2), 84.8 (SD: 13.2), and 6.4 (SD: 3.5), respectively. We also observed that there were many participants with abnormal WC (59.5%), HDL-C (51.4%), and SBP (58.4%). However, there were few participants with abnormal TG (20.5%) and DBP (43.2%). The participants with MetS had significantly higher abnormal WC (64.5% vs. 35.5%), TG (81.6 vs. 18.4%), HDL-C (61.1 vs. 38.9%), SBP (50.9 vs. 49.1%), DBP (67.5 vs. 32.5%), and FBG (64.1 vs. 35.9%) compared to those without.

**Table 3:** The distribution of South African minibus taxi drivers by components of MetS.

	**Entire cohort (*****n*** = **185)**		**IDF MetS**				
	**Mean** ±**SD**	***n*** **(%)**	**No MetS (77)**		**With MetS (108)**		***P*** **value “between groups”**
			**Mean** ±**SD**	***n*** **(%)**	**Mean** ±**SD**	***n*** **(%)**	
Waist circumference (cm)	99.1 ± 18.3		90.7 (14.5)		110.8(16.7)		< 0.001
Normal < 94		75 (40.5)		69 (92.0)		6(8.0)	
Abnormal ≥94		110 (59.5)		39 (35.5)		71(64.5)	
Triglycerides (mmol/l)	1.3 ± 1.1		1.0 (0.4)		1.9(1.5)		< 0.001
Normal < 1.7		147 (79.5)		101 (68.7)		46(31.3)	
Abnormal ≥1.7		38 (20.5)		7 (18.4)		31(81.6)	
HDL-C (mmol/l)	1.1 ± 0.3		1.2 (0.4)		1.0(0.3)		< 0.001
Normal ≥1.0		90 (48.6)		71 (78.9)		19(21.1)	
Abnormal < 1.0		95 (51.4)		37 (38.9)		58(61.1)	
Systolic blood pressure (mmHg)	133.4 ± 17.2		127 (13.3)		141(18.8)		0.002
Normal < 130		77 (41.6)		55 (71.4)		22(28.6)	
Abnormal ≥130		108 (58.4)		53 (49.1)		55(50.9)	
Diastolic blood pressure (mmHg)	84.8 ± 13.2		79 (9.1)		92.7(13.9)		< 0.001
Normal < 85		105 (56.8)		82 (78.1)		23(21.8)	
Abnormal ≥85		80 (43.2)		26 (32.5)		54(67.5)	
Fasting blood Glucose (mmol/l)	6.4 ± 3.5		5.3 (1.1)		7.9(4.8)		< 0.001
Normal < 5.5		93 (50.3)		75 (80.6)		18(19.4)	
Abnormal ≥5.5		92 (49.7)		33 (35.9)		59(64.1)	

HDL-C, high density lipoprotein cholesterol; IDF, international diabetes federation; MetS, metabolic syndrome.

Seventy-seven (*n* = 77) study participants met the IDF diagnostic criteria for MetS (i.e., had a clustering of 3 or more metabolic disorders), of which 46 had three (3) risk factors, 25 had four (4) risk factors, and 6 had five (5) risk factors. The distribution is shown in Table 4.

**Table 4:** The distribution of South African minibus taxi drivers by the clustering of MetS components.

**Number of metabolic disorders**	***n* (%)**
0	22 (11.9)
1	40 (21.6)
2	46 (24.9)
3	46 (24.9)
4	25 (13.5)
5	6 (3.2)

Metabolic disorders (abnormal WC, TG, SBP, DBP, HDL-C and FBG).

Table 5 presents the lifestyle practices based on the outcomes of MetS. Overall, the participants smoked an average of almost 10 cigarettes (SD: 5.3) a day, slept an average of 6.1 h (SD: 1.1) each day, spent an average of ZARR 92.1 (exchange rate: ZARR 1 = United States Dollar [USD]$ 17.23) (SD: 36.7) on SF each day, and had an average PAL of 1.42 MET-minutes per week (SD: 0.14). While there were no significant differences regarding the average number of cigarettes smoked by the participants or the average amount of money spent on SF between those who had MetS and those without MetS.

**Table 5:** The distribution of the South African minibus taxi drivers by their lifestyle risk factors (i.e., cigarettes smoking, alcohol consumption, physical activity level, sleep duration and money spent on street food each day) and MetS.

		**IDF metabolic syndrome**		
	**Entire cohort (*****n*** = **185)**	**No (*****n*** = **108)**	**Yes (*****n*** = **77)**	***P*** **value**
	***n*** **(%)**	***n*** **(%)**	***n*** **(%)**	***n*** **(%)**
**Cigarettes smoking**				
Yes	80 (43.2)	54 (50.0)	26 (33.8)	
No	105 (56.8)	54 (50.0)	51 (66.2)	0.028
**Average number of cigarettes smoked each day**				
M ± SD	9.9 ± 5.3	9.3 ± 4.9	11.0 ± 5.9	0.179
1–5	24 (30.0)	17 (31.5)	7 (26.9)	0.793
6-9	7 (8.8)	4 (7.4)	3 (11.5)	
≥10	49 (61.3)	33 (61.1)	16 (61.5)	
**Current alcohol drinking**				
Yes	100 (54.1)	55 (50.9)	45 (58.4)	0.312
No	85 (45.9)	53 (49.1)	32 (41.6)	
**Frequency of alcoholic beverage consumption**				
Monthly or less	52 (36.9)	31 (36.9)	24 (37.0)	0.248
2–4 time a month	51 (34.2)	30 (35.7)	21 (32.3)	
2–3 times a week	28 (18.8)	12 (14.3)	16 (24.6)	
4 or more time a week	15 (10.1)	11 (13.1)	4 (6.2)	
**Number of alcoholic beverages consumed on a typical day**				
1 or 2	9 (9.0)	4 (7.3)	5 (11.1)	0.906
3 or 4	9 (9.0)	4 (7.3)	5 (11.1)	
5 or 6	59 (59.0)	34 (61.8)	25 (55.6)	
7,8 or 9	18 (18.0)	10 (18.2)	8 (17.8)	
10 or more	5 (5.0)	2 (5.5)	2 (4.4)	
**Sleeping duration (hours)**				
M ± SD	6.1 ± 1.1	6.1 ± 1.0	6.2 ± 1.2	0.624
*n* (%)				
< 6	112 (60.5)	70 (64.8)	42 (54.5)	
≥7	73 (39.5)	38 (35.2)	35 (45.5)	0.159
**Money spend on street food each day (ZAR)**				
M ± SD	92.1 ± 36.7	88.6 ± 37.7	96.9 ± 34.9	0.056
*N* (%)				
< R99.00	96 (52.7)	63 (60.0)	33 (42.9)	
≥R100.00	86 (47.5)	42 (40.0)	44 (57.1)	0.022
**Physical activity level (MET-minutes per week)**				
*N* ± SD	1.42 ± 0.14	1.35 ± 0.12	1.51 ± 0.10	< 0.001
**Sedentary PAL**				
< 1.4	94 (50.8)	33 (30.6)	61 (79.2)	
**Low PAL**				
1.4-1.69	86 (46.5)	75 (69.4)	11 (14.3)	
Moderate PAL				< 0.001
1.70-1.99	5 (2.7)	-	5 (6.5)	
**Vigorous PAL**				
≥2	-	-	-	

IDF, International Diabetes Federation; ZAR, South African Rand (exchange rate 17.23 United States Dollar); PAL, physical activity level.

In terms of participant lifestyle distribution based on the MetS status, while there were no significant differences between participants with and without MetS for lifestyle practices such as alcohol consumption and sleeping duration, there were significantly higher number of nonsmokers who were positive for MetS (those who gave an affirmative response for smoking) and those who were negative for MetS (those who gave a negative response for smoking). There were also significantly more participants with MetS who spent ZARR 100 or more than those who spent less than 100 ZAR (57.1% vs. 42.9%, p=0.022). Finally, there were significantly more sedentary participants with MetS compared to those with low and moderate PAL (79.2% vs. 14.3% and 6.5%).

According to Table 6, smokers, those who spent ZARR 100 or more and those who spent < 1.4 MET-minute/week were 1.96, 2.0, and 13.6 times significantly more likely to suffer from MetS compared to those who did not smoke, those who spent less than ZARR 100, and those who spent 1.4 or more MET-minute/week. While the increased significant likelihood of MetS for sedentary activity remained, even after removing the confounding effects of sociodemographic characteristics and other lifestyle practices explored in the current study, the likelihood of smoking and the amount spent on SF disappeared. It is also important to note that removing the confounding effects of the other lifestyle facts of the participants resulted in an increased significant likelihood for developing MetS by 2.2 and 2.1 times for those who consumed alcohol and those who slept 7 h or more, respectively.

**Table 6:** Binary logistic regression analysis to show the association between the lifestyle factors and MetS status of the South African minibus taxi drivers.

	**IDF—metabolic syndrome**											
	**Crude**			**Model 1**			**Model 2**			**Model 3**		
	**OR**	**95% CI**	***P*** **value**	**AOR**	**95% CI**	***P*** **value**	**AOR**	**95% CI**	***P*** **value**	**AOR**	**95% CI**	***P*** **value**
**Current cigarettes smoking**												
No	1	1.072–3.590	0.029	1	0.301–1.063	0.077	1	0.318–1.142	0.120	1	0.270–1.208	0.143
Yes	1.962			0.566			0.602			0.571		
**Alcohol consumption**												
No	1			1			1			1		
Yes	1.355	0.751–2.444	0.312	1.776	0.935–3.374	0.079	1.706	0.886–3.287	0.110	2.191	1.021–4.699	0.044
**Sleeping duration**												
< 6	1			1			1			1		
≥7	1.535	0.844–2.791	0.160	1.558	0.832–2.915	0.166	1.497	0.786–2.851	0.220	2.107	0.977–4.521	0.057
**Money spends on street food each day (ZAR)**												
< R99.00	1			1			1			1		
≥R100.00	2.000	1.101–3.633	0.023	1.225	0.619–2.423	0.560	1.400	0.691–2.838	0.350	1.157	0.548–2.441	0.702
**PAL (MET–minutes per week)**												
Active (low, moderate and vigorous) PAL≥1.4	1	6.388–29.109	< 0.001	1	5.449–29.354	< 0.001	1	5.769–32.780	< 0.001	1	6.769–36.891	< 0.001
Sedentary (< 1.4)	13.636			12.647			13.751			15.802		

IDF, International Diabetes Federation; ZAR, South African Rand (exchange rate 17.23 United States Dollar); PAL, physical activity level; OR, odds ratio; AOR, adjusted odds ratio = Model 1: adjusted for age and driving experience; Model 2: adjusted for age, ethnicity, level of education, marital status and driving experience; Model 3: adjusted for tobacco smoking, alcohol consumption, sleeping duration, PAL and money spent on street food each day.

The frequency of SF consumption in relation to the likelihood of developing MetS and its components was also analyzed and is presented in Supplementary Table 1. Approximately 40.0% of the entire population consumed processed meat (sausages, polony, and cold cuts Viennas, Frankfurters, Russians, and salami) at least 1–3 times a week, with a significantly higher proportion (44.4%) of them experiencing abnormal BP compared to those with normal BP (37.5%). Similar results were also observed for the participants who consumed takeaway foods. Moreover, a higher proportion of participants with MetS and hypertension consumed fried food and snacks (i.e., chips, vetkoek, fried chicken, fried fish) compared to those who did not consume these foods. The daily consumption of deep-fried foods was also associated with an abnormal WC.

As illustrated in Table 7, consuming processed meat daily increases the risk of abnormal HDL-C by 3.7 times, while avoiding processed meat reduces hypertension. Further, avoiding takeout reduced the likelihood of developing MetS by 68.2% and abnormal TG by 25%. Daily takeout meal consumption increased hypertension risk by 3.1 times, even after adjusting for age and sociodemographic charactristics. The daily consumption of fried meat and fish increased the likelihood of developing MetS, abnormal WC, and hypertension by 2.1, 2.2, and 2.3 times, respectively. The association remained unchanged, even after removing the confounding effects of age, ethnicity, money spent on buying these foods, sociodemographic characteristic, and unhealthy lifestyle practices.

**Table 7:** The logistic regression analysis to show the association between the street foods consumed by the South African minibus taxi drivers and their MetS status.

	**MetS**		**Abnormal WC**		**Abnormal FBG**		**Abnormal HDL–C**		**Hypertension**		**Abnormal triglyceride**	
**Frequency of food consumption**	**OR**	**95% CI**	**OR**	**95% CI**	**OR**	**95% CI**	**OR**	**95% CI**	**OR**	**95% CI**	**OR**	**95% CI**
**Processed meat (sausages, polony, and cold cuts Viennas, Frankfurters, Russians, salami)**												
None	0.963	0.416–2.227	1.024	0.477–2.199	0.842	0.390–1.819	1.056	0.477–2.376	0.413*	0.182–0.937	1.165	0.459–2.957
Every day	1.143	0.318–4.109	0.594^$^	0.208–1.702	0.605^$^	0.205–1.783	3.667^*#°@*$*∧^	4.881–15.264	1.353	0.461–3.975	0.879	0.200–3.859
1–3 times last week	0.830	0.366–1.882	1.007	0.481–2.107	0.756	0.357–1.599	0.850	0.388–1.860	0.831	0.390–1.768	0.563	0.210–1.506
4–6 times last week	1		1		1		1		1		1	
**Takeaway food (pizza, burgers, chicken and fish parcels)**												
None	0.318*	0.115–0.878	1.618	0.266–9.852	0.865	0.163–4.602	1.250	0.173–9.019	0.767	0.075–7.860	0.750^*°@*$*^	1.574–67.602
Every day	1.227	0.263–5.734	1.014	0.448–2.298	1.300	0.583–2.899	0.833	0.338–2.052	3.097^*#$^	1.123–8.544	3.055	0.680–13.729
1–3 times last week	0.738	0.275–1.981	0.518	0.177–1.514	1.615	0.538–4.853	0.917	0.276–3.040	1.533	0.402–5.841	1.853	0.277–12.389
4–6 times last week	1		1		1		1		1		1	
**Fried meat and fish dishes (chips, vetkoek, fried chicken, fried fish)**												
None	1.778	0.236–13.405	1.200^$^	0.471–3.056	1.059	0.406–2.762	1.128	0.393–3.236	1.310	0.465–3.684	2.000	0.533–7.508
Every day	2.051^*#°@*$*∧^	1.757–5.558	2.223^*#°@∧^	1.007–4.904	1.310	0.595–2.882	1.974	0.851–4.580	2.278*	1.977–5.312	2.082	0.696–6.227
1–3 times last week	1.697	0.447–6.439	1.079	0.850	1.116	0.501–2.486	2.538^*#°@*$*∧^	1.089–5.918	0.776	0.314–1.916	1.358	0.437–4.228
4–6 times last week	1		1		1		1		1		1	
**Fried snacks (vetkoek, samoosas, doughnuts)**												
None	1.786	0.550–5.802	0.457	0.191–1.096	1.315	0.551–3.138	1.875	0.678–5.182	0.483	0.180–1.301	0.520	0.139–1.945
Every day	3.772^*°@^	1.479–9.616	1.760^#°^	0.693–4.470	1.004	0.431–2.342	2.333^*#^	2.880–6.188	2.194^*^	1.927–5.191	1.354	0.459–3.998
1–3 times last week	1.875	0.730–4.816	0.656	0.320–1.342	0.994	0.494–2.001	2.035	1.898–4.611	0.842	0.403–1.703	0.780	0.303–2.006
4–6 times last week	1		1		1		1		1		1	
**Packaged snacks (chips/crisps, mazimba)**												
None	3.036	0.593–15.547	0.606	0.186–1.977	0.643	0.201–2.052	3.354	0.716–18.637	0.381	0.117–1.241	3.056	0.351–26.593
every day	3.125	0.474–20.583	0.273	0.063–1.178	1.111	0.262–4.719	8.000^*#°@*$*∧^	1.215–52.693	0.889	0.216–3.662	3.300	0.294–37.103
1–3 times last week	4.113^*°@∧^	1.862–19.626	0.739	0.244–2.237	0.705	0.239–2.086	6.538^$^	1.373–31.132	0.660	0.226–1.926	3.075	0.379–24.933
4–6 times last week	1		1		1		1		1		1	
**Sugar Sweetened beverages (gas/fizzy cold drink and reconstituted)**												
None	0.545	0.133–2.236	0.541	0.186–1.577	0.923	0.317–2.685	0.426	0.127–1.427	0.500	0.127–1.965	0.967	0.229–4.087
Every day	1.660^*^	1.796–3.461	1.164	0.608–2.228	1.620	0.850–3.088	0.833	0.416–1.668	1.691	0.848–3.372	0.767	0.324–1.812
1–3 times last week	1.889	0.772–4.619	0.854	0.383–1.904	0.997	0.444–2.235	1.193	0.502–2.833	1.442	0.610–3.409	1.364	0.507–3.669
4–6 times last week	1		1		1		1		1		1	

FF, food frequencies; OR: ^*^p < 0.05; ^#^Model 1= AOR, p < 0.05 adjusted for age; °Model 2 = AOR, p < 0.05 adjusted for ethnicity; ^@^Model 3 = AOR, p < 0.05 adjusted for money spent on street food each day; ^$^Model 4 = AOR, p < 0,05 adjusted for all socio demographic status, ^∧^Model 5 = AOR, p < 0.05 adjusted for all lifestyle factors.

Moreover, the consumption of these foods 1–3 times a week increased the likelihood of developing abnormal HDL-C by 2.5 times, and this interaction also remained unchanged, even after removing all the confounding effects. The daily consumption of fried snacks also increased the likelihood of developing MetS, abnormal WC, abnormal HDL-C, and hypertension by 3.8, 1.7, 2.3, and 1.9 times, respectively. The consumption of packaged snacks such as crisps and amazimba (Niknaks Maize Snack) every day also increased the likelihood of abnormal HDL-C by eight times. Moreover, consuming these snacks 1–3 times a week increased the likelihood of developing MetS by 4.1 times. These interactions remained unchanged even after removing the confounding effects such as age, ethnicity, money spent on these foods, and sociodemographic status, and unhealthy lifestyle practices. The daily consumption of SSB increased the likelihood of developing MetS by 1.6 times. However, this interaction disappeared after removing the confounding effects such as age, ethnicity, money spent on these foods, sociodemographic characteristics, and unhealthy lifestyle practices.

## Discussion

The current study aimed to investigate the risk factors for MetS among the male minibus taxi drivers working in Cape Town and the surrounding areas. The majority of the taxi drivers had abnormal levels of WC, HDL-C, SBP, and FBG. Approximately 70% of the taxi drivers had clusters of three or more of these health issues. These results are corroborated by both national and international literature that show that individuals who are in the long-duration driving occupation, including taxi drivers, have a high likelihood of developing metabolic disorders compared to other professionals such as industrial and office workers (1–3, 27–30). In addition, these studies also identified age, driving duration, and driving experience as factors that accelerate the onset of these metabolic diseases (31–34). More importantly, Hildrum et al. (35) long argued that this condition strongly increases with age, regardless of any algorithm used to measure MetS. Indeed, in the current study, the mean age of the minibus taxi drivers was 40 years, with older participants having more driving experience compared to their younger counterparts. Even though the current analysis did not demonstrate a significant relationship between driving experience and MetS, it showed that old age increased the likelihood of developing MetS by up to 2.95 times.

Moreover, like in the current study, the majority of occupational drivers involved in other studies (28, 36) reported sleeping hours that are less than the recommended 6 h of sleep each day (37). This may be due to these drivers' long and irregular shift hours (28, 31). Unlike the aforementioned international researchers who reported sleep duration and its quality as the determinants of MetS, in the current study, the association between sleeping duration and MetS was significant. However, it is important to note that the majority of the minibus taxi drivers participating in the current research also reported long working hours such that their daily shifts started as early as 5 am most days and sometimes ended after 10 pm. They cite reasons such as the need to secure passengers who start work early and those who knock off late in the evenings from work because of their long working hours.

In the current study, despite no significant differences observed in the number of cigarettes smoked by those who had MetS and those without MetS, on average, the minibus taxi drivers smoked almost 10 cigarettes each day, and smoking increased their likelihood of developing MetS by up to two times. However, this interaction disappeared after we removed the confounding effects, such as sociodemography and other lifestyle practices investigated in the current study. Therefore, this suggests that factors such as age, ethnicity, the number of cigarettes smoked, and so on moderate propensity of smoking and the likelihood of developing MetS. Additionally, while some of the literature (32, 33) could not establish a relationship between smoking and the likelihood of developing MetS among occupational drivers, Appiah et al. (38) found that non-users of tobacco are less likely to suffer from MetS and its components. Mohebbi et al. (31) also showed that smokers are more likely to suffer from with MetS than nonsmokers. It is also important to explain the differences in the results regarding smokers between the current study and a recent study by Mabetwa et al. (32), which was also conducted for South African taxi drivers. Mabetwa et al.'s (32) study was conducted in the Gauteng province, while the current study was conducted in the Western Cape province. According to Statistics South Africa (39), Cape Town has the highest concentration of male smokers in South Africa. Additionally, it is commonly reported that smokers often smoke in public places, increasing the likelihood of exposure to secondhand smoke for nonsmokers. Therefore, we observed a high prevalence of MetS among nonsmokers in the current study. Moreover, it is important to highlight that the prevalence of smokers in the current study was higher than that of smokers reported in Mabetwa's study (43 vs. 30%).

In the current study, we also found results suggesting that minibus taxi drivers with a sedentary lifestyle had a 13-fold increased risk of developing MetS. This relationship remained strong even after removing the confounding effects such as sociodemography and other lifestyle factors investigated in the current study. This study indicates that physical activity has an independent and significant impact on metabolic health independently, regardless of other social determinants of health. These results are corroborated by substantiated evidence from international studies(40, 41) suggesting a significant negative correlation between physical activity and the likelihood of developing MetS among bus and taxi drivers. Moreover, Chen et al. (1) showed that sedentary occupations, including taxi driving, increase the risk of developing MetS. Several international studies have shown the dose–response relationship between physical activity and metabolic outcomes (13–18). According to Myers et al. (42), most active individuals generally have a low risk of developing metabolic diseases. Additionally, the aforementioned studies found that even meeting the minimal physical activity requirements outlined in the health guidelines (14) (i.e., at least 150 min per week of moderate-intensity activity or 75 min per week of vigorous activities) has significant benefits for reducing metabolic risk. However, we also have to acknowledge that, in our analysis, even though the participants who suffered from MetS had a higher PAL than those without MetS, their activity levels were still within the low PAL range (i.e., they were within the range of 1.4 and 1.7 Met-minutes per week). Thus, the average 1.51 MET-min per week dosage they obtained could not improve their metabolic health. We also must acknowledge that other studies could not find a significant association between physical activity and MetS (32, 38). The reason for this is currently unknown and needs further investigation.

Other interesting results from the current research were that the type and quality of food and beverages consumed by minibus taxi drivers impacted their metabolic health. For instance, when the confounding effects of other lifestyle factors were removed from the current study, alcohol consumption increased the risk of MetS by up to two times. Even though we did not measure the exact amount of alcohol consumed by the minibus taxi drivers participating in our research, the majority reported that they consumed alcoholic beverages that ranged from 5 to 9 standard drinks most days. This is a cause for concern given that studies by Hernández-Rubio et al. (43) and Fan et al. (44) found that heavy drinking is independently associated with reduced kidney function and metabolic risk factors such as impaired fasting glucose/diabetes mellitus, abdominal obesity, arterial stiffness and plaque buildup, hypertension, and dyslipidemia. In the current analysis, we also found that the consumption of takeaway foods, fried foods, and snacks such as crisps and SSB sold by the SF vendors increased the likelihood of developing MetS, abnormal HDL-C, TG, and hypertension. We also found that avoiding takeaway and fried foods decreased the likelihood of MetS.

International research by Kim and Je (45) corroborates our finding in that individuals with MetS generally consume large quantities of processed meat (such as sausages, polony, and cold cuts such as Viennas, Frankfurters, Russians, and salami). Furthermore, the aforementioned study also found that participants in the highest category of total meat, red meat, and processed meat consumption had an increased risk of developing MetS by approximately 14, 33, and 35%, respectively, compared to those in the lowest consumption category of these foods. A meta-analysis of studies (46, 47) revealed a strong correlation between the consumption of red meat and the likelihood of developing MetS after excluding studies from Asia. For instance, Pan et al. (48) found that even a slight increase in the daily consumption of red and processed meat had a 14% and 32% increase in the likelihood of type 2 diabetes mellitus, respectively. Abete et al. (49) also found high rates of mortality due to metabolic disorders in populations with high consumption of processed meat.

Some potential mechanisms have been explained to indicate the association between processed meat consumption and the likelihood of developing MetS. Among these are the findings that total and saturated fat contained in processed meat increase the risk of MetS through increased body fat centralization, hyperinsulinemia, and hyperglycemia, which are important components of MetS (50). According to Abete et al. (49), the aforementioned mechanism is mediated by nitrosamines. This chemical is toxic to pancreatic cells formed from the nitrates used as preservatives in processed meat. Additionally, these compounds cause insulin resistance.

Moreover, Marku et al. (51) argue that because iron is a strong pro-oxidant, it causes oxidative stress, which can harm tissues such as pancreatic beta cells. Furthermore, the aforementioned researchers argue that high iron levels may inhibit glucose metabolism and reduce pancreatic insulin synthesis and secretion. Based on the literature, we must also acknowledge that high levels of inflammatory mediators, such as C-reactive protein, in people who consume a high amount of red and processed meat could be another reason for the increased risk of MetS. Because C-reactive proteins also increase blood pressure (52), this could explain the association we found in the current research between the consumption of processed meat and hypertension. Griep et al. (53) reported similar results that suggest high consumption of processed meat is positively associated with the risk of hypertension. Another possible explanation for our findings may be those given by Micha et al. (54), who suggest that the high sodium content of processed meat results in elevated blood pressure.

Our current study further found the association between MetS risk and high consumption of fried food bought from street vendors and consumed on-site (i.e., fries/chips, vetkoek, fried chicken, and fried fish, to be specific). Our results were unsurprising given that the food sold on South African streets, including at the transport interchange areas where we recruited our participants, is not healthy. Additionally, Mchiza et al. (8) and Flores et al. (55) showed that, besides fruits and vegetables, most of the SF sold by vendors are not healthy as they are deep-fried, which is associated with cardiovascular risks. However, we must acknowledge that not all researchers have found associations between fried food and the risk of MetS. For instance, upon investigating a Mediterranean cohort of young Asian adults, Sayon-Orea et al. (56) and Kang and Kim (57) found no association between MetS and the frequency of consuming fried foods. The differences between the results of our study and those of the aforementioned Asian studies could be based on the type of food groups included in the current study and the two Asian studies; among the four groups of fried food included in the Asian studies were fried vegetables, fried fish, and fried seaweed. Therefore, we must always be cognizant of the literature that associates plant foods and fish with preventing metabolic diseases (58, 59). In the current study, on the other hand, the four groups of fried food were deep-fried potato chips (or French fries), vetkoek (a cake of deep-fried dough that is stuffed inside), fried chicken, and fried fish. In this case, fried vegetables and seaweeds impact health differently than fried potatoes and fried starch. Finally, the frying mechanisms in these studies were also different. In Asia, pan frying is mostly preferred, while deep frying is favored in South Africa, and these cooking methods have also been shown to impact health differently (60, 61).

This study's results found a statistically significant association between fast-food consumption and MetS risk. These results are consistent with those of Bahadoran et al. (62), where they showed evidence that regular fast-food consumption has a detrimental effect on general health and can increase the risk of obesity, insulin resistance, and other metabolic abnormalities. Several mechanisms have been proposed to explain the negative effects fast foods have on health outcomes. One such mechanism is that fast foods are energy-dense, thus modulating the weight gain process (63). Indeed, Mchiza et al. (8) showed that most fast foods sold in the streets of South Africa are energy dense and have an energy density that is almost two times the recommended energy for a healthy meal. Moreover, the mean total energy of these meals is estimated to be approximately 158–163 kcal per 100 g of food, with the total fat percent of beef hamburgers, chips, chicken hamburgers, and hot dogs being reported to be about 35.8 ± 10.7, 35.8 ± 8.7, 23.0 ± 5.1, and 34.0 ± 13.5%, respectively, with most of this fat being saturated fat (64).

The current study also showed that consuming packaged snacks (chips/crisps and mazimba) 1–3 times a day was associated with an increased risk of developing MetS. In agreement with this study's results, a significant relationship was also shown between dyslipidemia and the frequency of consumption of hydrogenated fat, fast foods, cheese puffs, and crisps in both urban and rural areas of Iran (65).

The current analysis also showed that the consumption of SSBs increased the risk of MetS by up to 1.8 times. Consistent with this study's are a few international studies (66, 67) that reported that SSB intake has significant effects on MetS risk. Moreover, a study conducted on 596 young adult South Africans by Seloka et al. (68) also showed that high consumption of SSBs increases the risk of high FBG in men. This is not a surprise since Deshpande et al. (67) have long shown that sweetened beverages disrupt the hormones involved in regulating energy balance and the satiety center within the human limbic system, which may lead to overeating and result in an increase in positive energy balance in the body. Therefore, the results are body weight gain and an increase in WC. It is also important to note that the SSBs that were included in our study consisted of cold drinks and reconstituted gas/fizzy drinks. Overconsumption of fructose and sucrose from these SSBs has been shown to stimulate and initiate lipid production in the liver, resulting in higher serum triglyceride and cholesterol levels, visceral fat accumulation, and plaque buildup (69). Moreover, glucose in SSBs has a higher glycaemic index, which can cause high blood glucose spikes and may lead to glucose intolerance, insulin resistance, and an increase in inflammatory biomarkers (70).

Finally, in the current study, we found that avoiding the consumption of fresh fruits increased the likelihood of developing abnormal HDL-C. Although we could not specify the type, color, and amount of fruit we referred to in our research, we could attribute these significant interactions to the fiber and antioxidants that fresh fruit and vegetables have, which are compounds that have been shown to mitigate metabolic risks (71). Although several epidemiological studies have evaluated the association between fruit and vegetable consumption and the risk of MetS, the results remain controversial. For instance, some studies have emphasized fruits' and vegetables' roles in mitigating metabolic disease risk or eliminating the disease entirely (72–74), while others have shown the opposite or no association at all with disease downregulation (75). However, a meta-analysis of international studies by Tian et al. (76) cleared up the controversy by showing that, when data from these studies were combined, high fruit and vegetable consumers were 13% and 24% less likely to have MetS, respectively. This meta-analysis of 78 studies further investigated the relationship between the consumption of fruits, vegetables, and MetS risk. When these researchers stratified these interactions by continent, the inverse association of fruit and vegetable consumption was observed to be OR: 0·86 (0·77, 0·96) and OR: 0·86 (0·80, 0·92), respectively, with the risk of MetS remaining significant in Asia. Based on these results, they concluded that people should consume more fruits and vegetables to reduce the risk of metabolic diseases. However, we should be cognizant of the amount, type, and quality of fruit and vegetables that bring about this kind of health benefit. Studies by Nguyen et al. (77) and Sharma et al. (78) suggest that plant foods high in fiber, such as brown and white rice, have greater metabolic health benefits. Numerous substantiated pieces of evidence suggest that the consumption of good fatty acids can prevent MetS risk and its components (79–83). Sekgala et al. (81), in their recent research, eloquently indicated that substituting SFA for PUFA significantly decreases the likelihood of elevated BP by 7%.

To end the aforementioned arguments, it is also important to highlight that, unlike many studies conducted in South African populations with financial constraints, the current study was based on a population that could afford to procure food. Hence, the majority spent more than ZARR 100 on buying SF. A hundred ZAR and more a day is way above the recommended amount (ZARR 40 per day) per person recommended as enough budget to spend on healthy food each day. Abraham et al. (84) have long suggested that, on average, for an adult South African man, a healthier diet costs ZARR 17.3 (which is about USD$ 1) per day. In the current research, the minibus taxi drivers who spent more than ZARR 100 on SF had two times greater risk of MetS than their counterparts who spent ZARR 99 or less. This adverse outcome of MetS could be attributed to the unhealthy food options readily available near transportation hubs. While this relationship was lifestyle and sociodemography dependent, the amount spent on SF was not found to be the mediator/moderator of the type of foods purchased and consumed by the minibus taxi drivers included in the current study.

## Limitation

Despite the notable and important results of the current study outlined above, there are a number of limitations to this study that need to be considered. First, the study was cross-sectional. Hence, causal inferences cannot be drawn from this study's results. Second, the results of the current study focused only on South African male taxi drivers. Therefore, they can only be generalized to occupational drivers in the long-duration driving business but not to the general population. Finally, even though most of the major confounders have been taken into account in most of the studies, there is still a chance of unmeasured and residual confounding factors impacting in the current study. The confounders that were taken into account in the current study were also different from those in the other international studies that have been used to corroborate/contrast this study's results. Hence, notable differences were observed.

## Conclusion

In the current study, we have shown the significant determinants of MetS and its components among South African minibus taxi drivers who presented with abnormal levels of WC, HDL-C, SBP, and FBG, of whom 70% were diagnosed to have MetS according to the IDF diagnostic criteria. Among these important determinants of MetS, we showed that sociodemographic factors such as age and high experience in taxi driving are significantly associated with MetS risk and its components. Moreover, lifestyle factors such as fewer sleeping hours, smoking many cigarettes each day, alcohol and SSB consumption, spending a lot of money on SF, and being sedentary impacted the minibus taxi drivers' metabolic health. More importantly, the consumption of fried food, processed foods, and commercially packaged snacks like crisps, obtained as takeaways, increased the likelihood of minibus taxi drivers developing MetS and its components. However, avoiding the consumption of takeaway and fried foods reduces the risk of MetS. Finally, avoiding the consumption of fruit increased MetS risk. These results have significant public health implications, as policymakers need to adopt evidence-based strategies to encourage a healthy lifestyle among South African men, especially minibus taxi drivers.

## Data availability statement

The original contributions presented in the study are included in the article/Supplementary material, further inquiries can be directed to the corresponding author.

## Ethics statement

The studies involving human participants were reviewed and approved by Ms. Patricia Josias Research Ethics Committee Officer University of the Western Cape. The patients/participants provided their written informed consent to participate in this study.

## Author contributions

MS and ZM: conceptualization and funding acquisition. MS: formal data analysis, methodology, and writing–original draft. ZM and MO: supervision, writing, review, and editing. BM: biochemical analysis. All authors contributed to the article and approved its submitted version.
