# Supplementary material for: Sociodemographic and lifestyle factors and the risk of metabolic syndrome in taxi drivers: A focus on street food
Source: Front Nutr. 2023 Feb 23;10:1112975. doi: 10.3389/fnut.2023.1112975 (PMC9996058; doi:10.3389/fnut.2023.1112975)
Supplement: Supplementary file 1 [file Table_1.docx]

Supplementary Material

Socio-demographic and lifestyle factors and the risk of metabolic syndrome in taxi drivers. A focus on street food

**Machoene Derrick Sekgala^1,2*,^ Maretha Opperman^3^, Buhle Mpahleni^3^ and Zandile June-Rose Mchiza^1,4^**

*** Correspondence:** Machoene Derrick Sekgala

dsekgala@hsrc.ac.za

# Supplementary Table

**Table S1:** The frequency of consuming street food by the metabolic status of South African minibus taxi drivers

|  |  | IDF MetS | | WC | | FBG | | HDL-C | | Hypertension | | Triglyceride | |
| --- | --- | --- | --- | --- | --- | --- | --- | --- | --- | --- | --- | --- | --- |
| FF | Entire cohort | absent | Present | normal | abnormal | normal | abnormal | normal | abnormal | normal | abnormal | normal | abnormal |
|  | n(%)  [95%CI] | n(%)  [95%CI] | n(%)  [95%CI] | n(%)  [95%CI] | n(%)  [95%CI] | n(%)  [95%CI] | n(%)  [95%CI] | n(%)  [95%CI] | n(%)  [95%CI] | n(%)  [95%CI] | n(%)  [95%CI] | n(%)  [95%CI] | n(%)  [95%CI] |
| **processed meat such as (sausages, polony, cold cuts Viennas, Frankfurters, Russians, salami** | | | | | | | | | | | | | |
| none | 62(33.9)  [27-41] | 36(33.3)  [25-43] | 26(34.7)  [25-46] | 25(33.3)  [24-45] | 37(34.3)  [26-44] | 30(33.0)  [24-43] | 32(34.8)  [26-45] | 31(34.4)  [25-45] | 31(33.3)  [24-44] | 45(37.5)  [29-47] | 17(27.0)*  [17-39] | 47(32.4)  [25-41] | 15(39.5)  [25-56] |
| every day | 13(7.10)  [4-12] | 7(6.5)  [3-13] | 6(8.0)  [4-17] | 7(9.3)  [4-18] | 6(5.6)  [2-12] | 8(8.8)  [4-17] | 5(5.4)  [2-12] | 2(2.2)  [1-9] | 11(11.8)  [7-20] | 7(5.8)  [3-12] | 6(9.5)*  [4-20] | 10(6.9)  [4-12] | 3(7.9)  [3-22] |
| 1-3 times last week | 73(39.9)  [33-47] | 45(41.7)  [33-51] | 28(37.3)^  [27-49] | 29(38.7)  [28-50] | 44(40.7)  [32-50] | 38(41.8)  [32-52] | 35(38.0)  [29-48] | 39(43.3)  [33-54] | 34(36.6)  [27-47] | 46(37.5)  [29-47] | 28(44.4)*  [33-57] | 62(42.8)  [35-51] | 11(28.9)  [17-45] |
| 4-6 times last week | 35(19.1)  [14-26] | 20(18.5)  [12-27] | 15(20)  [12-31] | 14(18.7)  [11-29] | 21(19.4)  [13-28] | 15(16.5)  [10-26] | 20(21.7)  [14-31] | 18(20.0)  [13-30] | 17(18.3)  [12-28] | 23(19.2)  [13-27] | 12(19.0)*  [11-31] | 26(17.9)  [12-25] | 9(23.7)  [13-40] |
| **Food from fast food outlets take-aways (pizza, chicken, fish)** | | | | | | | | | | | | | |
| none | 5(2.7)  [1-6] | 3(2.8)  [1-8] | 2(2.7)  [1-10] | 2(2.7)  [1-10] | 3(2.8)  [1-8] | 4(4.4)  [2-11] | 1(1.1)  [0-7] | 2(2.2)  [1-9] | 3(3.2)  [1-10] | 5(4.2)  [2-10] | 0(0.0)*  - | 2(1.4)  [0-5] | 3(7.9)  [3-22] |
| every day | 138(75.4)  [69-81] | 78(72.2)  [63-80] | 60(80)  [69-88] | 53(70.7)  [59-80] | 85(78.7)  [70-85] | 64(70.3)  [60-79] | 74(80.4)  [71-87] | 69(76.7)  [67-84] | 69(74.2)  [64-82] | 85(70.8)  [62-78] | 53(84.1)*  [73-91] | 107(73.8)  [66-80] | 31(81.6)  [66-91] |
| 1-3 times last week | 18(9.8)  [6-15] | 11(10.2)  [6-18] | 7(9.3)  [4-18] | 10(13.3)  [7-23] | 8(7.4)  [4-14] | 9(9.9)  [5-18] | 9(9.8)  [5-18] | 9(10.0)  [5-18] | 9(9.7)  [5-18] | 13(10.8)  [6-18] | 5(7.9)*  [3-18] | 15(10.3)  [6-17] | 3(7.9)  [3-22] |
| 4-6 times last week | 22(12)  [8-18] | 16(14.8)  [9-23] | 6(8.0)  [4-17] | 10(13.3)  [7-23] | 12(11.1)  [6-19] | 14(15.4)  [9-24] | 8(8.7)  [4-17] | 10(11.1)  [6-20] | 12(12.9)  [7-21] | 14(14.2)  [9-22] | 5(7.9)*  [3-18] | 21(14.5)  [10-21] | 1(2.6)  [0-17] |
| **Fried food bought from street vendors (chips, vetkoek, fried chicken, fried fish)** | | | | | | | | | | | | | |
| none | 22(12)  [8-18] | 14(13)  [8-21] | 8(10.7)*  [5-20] | 12(16.0)  [9-26] | 10(9.3)  [5-16] | 12(13.2)  [8-22] | 10(10.9)  [6-19] | 14(15.6)  [9-25] | 8(8.6)  [4-16] | 15(12.5)  [8-20] | 7(11.1)*  [5-22] | 16(11.0)  [7-17] | 6(15.8)  [7-31] |
| every day | 64(35)  [28-42] | 29(26.9)  [19-36] | 35(46.7)*  [36-58] | 17(22.7)  [15-34] | 47(43.5)  [34-53] | 28(30.8)  [22-41] | 36(39.1)  [30-50] | 30(33.3)  [24-44] | 34(36.6)  [27-47] | 33(27.5)  [20-36] | 31(49.2)*  [37-61] | 48(33.1)  [26-41] | 16(42.1)  [28-58] |
| 1-3 times last week | 64(35)  [28-42] | 40(37.0)  [28-47] | 24(32.0)*  [22-43] | 29(38.7)  [28-50] | 35(32.4)  [24-42] | 34(37.4)  [28-48] | 30(32.6)  [24-43] | 25(27.8)  [19-38] | 39(41.9)  [32-52] | 49(40.8)  [32-50] | 15(23.8)*  [15-36] | 52(35.9)  [28-44] | 12(31.6)  [19-48] |
| 4-6 times last week | 33(18)  [13-24] | 25(23.1)  [16-32] | 8(10.7)*  [5-20] | 17(22.7)  [15-34] | 16(14.8)  [9-23] | 17(18.7)  [12-28] | 16(17.4)  [11-27] | 21(23.3)  [16-33] | 12(12.9)  [7-21] | 23(19.2)  [13-27] | 10(15.9)*  [9-27] | 29(20.0)  [14-27] | 4(10.5)  [4-25] |
| **Food deep fried in oil/fat (fish, fries/chips, vetkoek, samoosas, doughnuts)** | | | | | | | | | | | | | |
| none | 27(14.8)  [10-21] | 18(16.7)  [11-25] | 9(12.0)  [6-22] | 16(21.3)  [13-32] | 11(10.2)*  [6-18] | 13(14.3)  [8-23] | 14(15.2)  [9-24] | 14(15.6)  [9-25] | 13(14.0)  [8-23] | 21(17.5)  [12-25] | 6(9.5)*  [4-20] | 24(16.6)  [11-24] | 3(7.9)  [3-22] |
| every day | 33(18)  [13-24] | 14(13.0)  [8-21] | 19(25.3)  [17-36] | 7(9.3)  [4-18] | 26(24.1)*  [17-33] | 14(15.4)  [9-24] | 19(20.7)  [14-30] | 14(15.6)  [9-25] | 19(20.4)  [13-30] | 15(12.5)  [8-20] | 18(28.6)*  [19-41] | 23(15.9)  [11-23] | 10(26.3)  [15-43] |
| 1-3 times last week | 90(49.2)  [42-56] | 57(52.8)  [43-62] | 33(44.0)  [33-55] | 41(54.7)  [43-66] | 49(45.4)*  [36-55] | 48(52.7)  [42-63] | 42(45.7)  [36-56] | 41(45.6)  [36-56] | 49(52.7)  [42-63] | 62(51.7)  [43-61] | 28(44.4)*  [33-57] | 72(49.7)  [42-58] | 18(47.4)  [32-63] |
| 4-6 times last week | 33(18)  [13-24] | 19(17.6)  [11-26] | 14(18.7)  [11-29] | 11(14.7)  [8-25] | 22(20.4)*  [14-29] | 16(17.6)  [11-27] | 17(18.5)  [12-28] | 21(23.3)  [16-33] | 12(12.9)  [7-21] | 22(18.3)  [12-26] | 11(17.5)*  [10-29] | 26(17.9)  [12-25] | 7(18.4)  [9-34] |
| **Fresh fruit (all the fruit, excluding fruit juices and dried fruit)** | | | | | | | | | | | | | |
| none | 26(14.2)  [10-20] | 15(13.9)  [9-22] | 11(14.7)  [8-25] | 12(16.0)  [9-26] | 14(13.0)  [8-21] | 11(12.1)  [7-21] | 15(16.3)  [10-25] | 9(10.0)  [5-18] | 17(18.3)  [12-28] | 18(15.0)  [10-23] | 8(12.7)  [6-24] | 19(13.1)  [8-20] | 7(18.4)  [9-34] |
| every day | 30(16.4)  [12-23] | 15(13.9)  [9-22] | 15(20.0)  [12-31] | 12(16.0)  [9-26] | 18(16.7)  [11-25] | 10(11.0)  [6-19] | 20(21.7)  [14-31] | 15(16.7)  [10-26] | 15(16.1)  [10-25] | 19(15.8)  [10-24] | 11(17.5)  [10-29] | 25(17.2)  [12-24] | 5(13.2)  [6-28] |
| 1-3 times last week | 89(48.6)  [41-56] | 53(49.1)  [40-59] | 36(48.0)  [37-59] | 34(45.3)  [34-57] | 55(50.9)  [41-60] | 49(53.8)  [43-64] | 40(43.5)  [34-54] | 43(47.8)  [38-58] | 46(49.5)  [39-60] | 57(47.5)  [39-57] | 32(50.8)  [39-63] | 72(49.7)  [42-58] | 17(44.7)  [30-61] |
| 4-6 times last week | 38(20.8)  [15-27] | 25(23.1)  [16-32] | 13(17.3)  [10-28] | 17(22.7)  [15-34] | 21(19.4)  [13-28] | 21(23.1)  [15-33] | 17(18.5)  [12-28] | 23(25.6)  [18-36] | 15(16.1)  [10-25] | 26(21.7)  [15-30] | 12(19.0)  [11-31] | 29(20.0)  [14-27] | 9(23.7)  [13-40] |
| **Snacks (chips/crisps, mazimba)** | | | | | | | | | | | | | |
| none | 45(24.6)  [19-31] | 28(25.9)  [18-35] | 17(22.7)  [15-34] | 20(26.7)  [18-38] | 25(23.1)  [16-32] | 24(26.4)  [18-36] | 21(22.8)  [15-33] | 26(28.9)  [20-39] | 19(20.4)*  [13-30] | 32(26.7)  [19-35] | 13(20.6)  [12-32] | 36(24.8)  [18-33] | 9(23.7)  [13-40] |
| every day | 13(7.1)  [4-12] | 8(7.4)  [4-14] | 5(6.7)  [3-15] | 8(10.7)  [5-20] | 5(4.6)  [2-11] | 5(5.5)  [2-13] | 8(8.7)  [4-17] | 5(5.6)  [2-13] | 8(8.6)*  [4-16] | 9(7.5)  [4-14] | 4(6.3)  [2-16] | 10(6.9)  [4-12] | 3(7.9)  [3-22] |
| 1-3 times last week | 113(61.7)  [54-69] | 82(57.4)  [48-66] | 51(68)  [57-78] | 42(56.0)  [45-67] | 71(65.7)  [56-74] | 56(61.5)  [51-71] | 57(62)  [52-71] | 49(54.4)  [44-64] | 64(68.8)*  [59-77] | 71(59.2)  [50-68] | 42(66.7)  [54-77] | 88(60.7)  [52-68] | 25(65.8)  [49-79] |
| 4-6 times last week | 12(6.6)  [4-11] | 10(9.3)  [5-16] | 2(2.7)  [1-10] | 5(6.7)  [3-15] | 7(6.5)  [3-13] | 6(6.6)  [3-14] | 6(6.5)  [3-14] | 10(11.1)  [6-20] | 2(2.2)*  [1-8] | 8(6.7)  [3-13] | 4(6.3)  [2-16] | 11(7.6)  [4-13] | 1(2.6)  [0-17] |
| **Sugar Sweetened beverages (gas/fizzy cold drink and reconstituted)** | | | | | | | | | | | | | |
| none | 14(7.7)  [5-13] | 11(10.2)  [6-18] | 3(4.0)  [1-12] | 8(10.7)  [5-20] | 6(5.6)  [2-12] | 9(9.9)  [5-18] | 5(5.4)  [2-12] | 10(11.1)  [6-20] | 4(4.3)  [2-11] | 11(9.2)  [5-16] | 3(4.8)  [2-14] | 11(7.6)  [4-13] | 3(7.9)  [3-22] |
| every day | 86(47)  [40-54] | 47(43.5)  [34-53] | 39(52.0)  [41-63] | 33(44.0)  [33-55] | 53(49.1)  [40-59] | 35(8.5)  [29-49] | 51(55.4)  [45-65] | 43(47.8)  [38-58] | 43(46.2)  [36-56] | 55(45.8)  [37-55] | 31(49.2)  [37-61] | 71(49.0)  [41-57] | 15(39.5)  [25-56] |
| 1-3 times last week | 35(19.1)  [14-26] | 18(16.7)  [11-25] | 17(22.7)  [15-34] | 15(20.0)  [12-31] | 20(18.5)  [12-27] | 20(22.0)  [15-32] | 15(16.3)  [10-25] | 14(15.6)  [9-25] | 21(22.6)  [15-32] | 21(17.5)  [12-25] | 14(22.2)  [14-34] | 25(17.2)  [12-24] | 10(26.3)  [15-43] |
| 4-6 times last week | 48(26.2)  [20-33] | 32(29.6)  [22-39] | 16(21.3)  [13-32] | 19(25.3)  [17-36] | 29(26.9)  [19-36] | 27(29.7)  [21-40] | 21(22.8)  [15-33] | 23(25.6)  [18-36] | 25(26.9)  [19-37] | 33(27.5)  [20-36] | 15(23.8)  [15-36] | 38(26.2)  [20-34] | 10(26.3)  [15-43] |
| **Tinned fish (sardines/pilchards/salmon (excluding tuna))** | | | | | | | | | | | | | |
| none | 90(49.2)  [42-56] | 52(48.1)  [39-58] | 38(50.7)  [39-62] | 38(50.7)  [39-62] | 52(48.1)  [39-58] | 42(46.2)  [36-57] | 48(52.2)  [42-62] | 49(54.4)  [44-64] | 41(44.1)  [34-54] | 61(50.8)  [42-60] | 29(46.0)  [34-58] | 73(50.3)  [42-58] | 17(44.7)  [30-61] |
| every day | 2(1.1)  [0-4] | 1(0.9)  [0-6] | 1(1.3)  [0-9] | 0 | 2(1.9)  [0-7] | 1(1.1)  [0-8] | 1(1.1)  [0-7] | 0 | 2(2.2)  [1-8] | 2(1.7)  [0-6] | 0 | 2(1.4)  [0-5] | 0 |
| 1-3 times last week | 81(44.3)  [37-52] | 52(48.1)  [39-58] | 29(38.7)  [28-50] | 36(48.0)  [37-59] | 45(41.7)  [33-51] | 42(46.2)  [36-57] | 39(42.4)  [33-53] | 36(40.0)  [30-51] | 45(48.4)  [38-59] | 53(44.2)  [35-53] | 28(44.4)  [33-57] | 65(44.8)  [37-53] | 16(42.1)  [28-58] |
| 4-6 times last week | 10(5.5)  [3-10] | 3(2.8)  [1-8] | 7(9.3)  [4-18] | 1(1.3)  [0-9] | 9(8.3)  [4-15] | 6(6.6)  [3-14] | 4(4.3)  [2-11] | 5(5.6)  [2-13] | 5(5.4  [2-12] | 4(3.3)  [1-9] | 6(9.5)  [4-20] | 5(3.4)  [1-8] | 5(13.2)  [6-28] |

MetS, metabolic syndrome; FF, food frequencies; WC, waist circumference; HDL-C, high density lipo-protein cholesterol; FBG, fasting blood glucose; * p<0.05

**
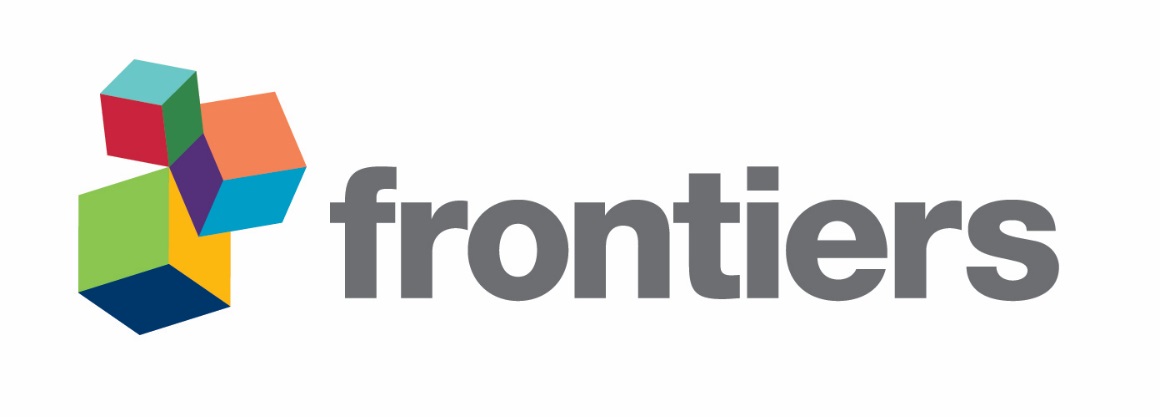
**
